# An objective assessment of toddlers’ physical activity and sedentary levels: a cross-sectional study

**DOI:** 10.1186/s12889-015-2335-8

**Published:** 2015-09-26

**Authors:** Leigh M. Vanderloo, Patricia Tucker

**Affiliations:** Health and Rehabilitation Sciences, Faculty of Health Sciences, Western University, 1201 Elborn College, Rm 2585, London, ON N6G 1H1 Canada; School of Occupational Therapy, Faculty of Health Sciences, Western University, 1201 Elborn College, Rm 2547, London, ON N6G 1H1 Canada

**Keywords:** Movement, Sedentary time, Young children, Accelerometer

## Abstract

**Background:**

Little evidence exists on the physical activity and sedentary time of Canadian toddlers; this study objectively measured such behaviors and compared participants’ activity levels to national guidelines. Levels of screen-viewing among toddlers were also explored.

**Methods:**

Forty toddlers (mean age = 25.7 months) wore Actical accelerometers for seven consecutive days (15 s epoch). Parents/guardians completed a wear-time log and a demographic and screen-viewing questionnaire. Descriptive analyses were used to determine participants’ levels of physical activity and sedentary time, to identify whether toddlers were meeting physical activity/sedentary guidelines, and to explore demographic variables. *T*-tests were used to assess whether toddlers’ activity levels differed based on cut-points applied and various demographic and screen-related variables. Regression analyses were conducted to examine associations between toddlers’ sedentary time and screen-viewing levels.

**Results:**

Toddlers engaged in 37.27 (*SD* = 3.79) to 49.40 (*SD* = 3.29) mins/hr of sedentary time, 9.79 (*SD* = 2.90) to 18.78 (*SD* = 3.22) mins/hr of light-intensity physical activity (LPA), 0.82 (*SD* = 0.72) to 3.95 (*SD* = 1.93) mins/hr of moderate- to vigorous-intensity physical activity (MVPA), and 10.60 (*SD* =3.29) to 22.73 (*SD* = 3.97) mins/hr of total physical activity (TPA), based on the Trost *et al.* and the Canadian Health Measures Survey (CHMS) cut-points respectively; these rates were significantly different (*p* <.001). On at least 1 day, 17.5 % (Trost *et al.* cut-points) and 97.5 % (CHMS cut-points) of the sample met or exceeded the Canadian physical activity guidelines. No statistically significant differences in sedentary time or physical activity (all intensities) based on sex were reported (*p* <.001); however, LPA (CHMS cut-points) did significantly differ based on childcare attendance (*p* <.05). Approximately 93.2 % of participants watched television, and 56.8 % utilized computers. Only 18.8 and 25.0 % of children under 2 years and 70.8 and 62.5 % of 2–3 years olds met the screen-use recommendation of the sedentary behavior guidelines on weekdays and weekend days, respectively.

**Discussion and conclusion:**

The implications of this work suggest that a greater understanding of toddlers’ activity patterns is needed; additional mechanisms of promoting active behaviors among this group should be explored.

## Background

Physical activity plays a pivotal role in the overall health and well-being of children. Among young children under the age of 5 years, regular physical activity has been linked to decreases in cardiovascular risk [1] as well as improvements in motor development [2], and psychosocial and cognitive factors [3]. Unfortunately, and based on recently published literature, there are considerable variability in the prevalence estimates of young children’s measured physical activity [4–6]. In fact, over the past decade, a great deal of research has focused on the physical activity and sedentary levels of preschoolers (i.e., 2.5–5 years) [2, 4, 5]. Interestingly, investigations into the physical activity and sedentary behaviors of toddlers (i.e., 18–29 months) are limited. In actuality, only a small number of studies have been conducted to examine their physical activity behaviors, where one relied on parent proxy report [7], two on direct observation [8, 9], and four on objective measures [10–14], where the single Canadian study assessed toddlers’ physical activity and sedentary levels during childcare hours only [13].

The Canadian Society of Exercise Physiology [15, 16] released physical activity and sedentary behavior guidelines for young children. Consistent with other international recommendations [17, 18], these guidelines stipulate that children between the ages of 1– 4 years should accrue a minimum of 180 min of physical activity (at any intensity) per day [15], and spend no more than 60 min at a time seated or restrained [16]. With regard to screen viewing, the Canadian sedentary behavior guidelines [16] suggest that children under the age of 2 should not engage in any screen time, and those 2–4 years should be limited to less than 1 h per day. However, the literature has yet to address the degree to which Canadian toddlers are meeting (or failing to meet) these recommendations. Moreover, little attention has been paid to the sedentary behaviors of toddlers in spite of the evidence suggesting that the majority of young children’s waking hours are spent being inactive [19, 20] and in front of screens [21–23], thus placing them at risk for developmental delays and poorer overall health status [21]. Given these gaps in the literature, additional attention is required to improve our understanding of Canadian toddlers’ activity patterns and behaviors.

Accelerometers represent one popular method for objectively measuring levels of physical activity and sedentary time among young children [14, 24, 25], and may prove useful in determining the activity levels of this age group. However, recent evidence suggests that the use of different accelerometer models and their respective cut-points make gaining an accurate understanding of young children’s physical activity levels challenging [26]. Consequently, data examining the difference in activity levels reported using various thresholds may be warranted to help inform the selection and application of toddler-specific cut-points.

This exploratory study sought to objectively measure the physical activity levels and sedentary time of a sample of toddlers in London, Canada using two sets of cut-points in comparison to the national physical activity guidelines. Because a variety of demographic variables have been identified as influencing young children’s activity levels, the impact of sex [27], parental education [28], annual family income [29], screen-viewing [30], and childcare enrollment [31] on toddlers’ physical activity and sedentary time were reported. Differences in physical activity and sedentary time accumulated on weekdays and weekend days were also examined [27]. Finally, this study aimed to explore toddlers’ screen-viewing (i.e., time spent engaged in these activities, weekend versus weekend day variation), and the proportion of participants that met/failed to meet the screen use portion of national sedentary behavior guidelines. Overall, we hypothesize that toddlers will accumulate high levels of sedentary time and low levels of physical activity. We also anticipate finding that this cohort will engage in high levels of screen-viewing activities.

## Methods

### Study sample & recruitment

Using a cross-sectional study design, English-speaking toddlers (between the ages of 18–29 months) from London, Canada were invited to participate. In an effort to target a geographically-representative sample, parents/guardians of participants were recruited at a mother and child exhibition, at various playgroups offered by the Ontario Early Years Centers (spanning various socio-economic areas), and via posters placed in locations frequented by parents/guardians and young children (e.g., all public libraries, Ontario Early Years Centers, childcare facilities, etc.). Where appropriate, snowball sampling was also utilized as a means of maximizing the reach of our recruitment methods.

### Study protocol

Data collection occurred between August 2013 and November 2014 (and ceased during the winter months to avoid seasonality effects). Participants were asked to wear an accelerometer for seven consecutive days (i.e., 5 weekdays and 2 weekend days; Monday to Sunday) during all waking hours; parents/guardians were asked to fit their child with the device upon them waking in the morning, and to remove it prior to their bedtime. In addition, parents/guardians were asked to keep a log of the on/off times of the accelerometers. Accelerometers and logs were dropped off to participants’ parents/guardians a few days prior to the first day of data collection (i.e., on Friday, Saturday, or Sunday, with data collection commencing on Monday). Following the week of data collection, a researcher returned to the participants’ homes to collect the accelerometers and logs. Ethical approval for the study protocol and related documents was obtained from the Office of the Research Ethics Board at the University of Western Ontario. Written informed consent was provided by parents/guardians of all participating children.

### Measurement

#### Toddler’s sedentary time and physical activity

Toddlers’ sedentary time and physical activity levels (i.e., light-intensity physical activity [LPA], moderate- to vigorous-intensity physical activity [MVPA], total physical activity [TPA]) were measured using Actical™ (MiniMitter, Bend, Oregon) accelerometers. These lightweight omnidirectional motion sensors provide detailed data on the duration and intensity of the children’s movements [14]. A 15-second epoch length was applied to capture the sporadic activity and intermittent periods of rest of the young participants [25]. Accelerometers were secured to the participants’ right hip using an adjustable belt and were programmed to begin collecting activity data on the morning (i.e., 6 am) of the first day of data collection (i.e., Monday). Participants (and their parents/guardians) were blind to all activity data collected while wearing the monitor.

#### Toddlers’ screen-viewing behaviors

Parents/guardians completed the Toddler Screen-Viewing Questionnaire. Informed by the work of Colley *et al.* [6], Certain and Khan [32], Vanderwater *et al.* [22], and Zimmerman *et al.* [23], this tool was created by the researchers to collect data on participants’ screen-viewing. Such items included whether the child used screens and which ones (e.g., yes/no; television, computer [i.e., laptops, tablets, smartphones], etc.), the amount of time spent engaged in screen-viewing activities per weekday and weekend day (presented in ranges and in line with Canada’s sedentary behavior guidelines [16]; i.e., no television/screen use, less than 30 min, 30–59 min, 60–89 min, 90–120 min, more than 120 min), reasons for engaging in screen-viewing activities (check all that apply; i.e., for education/entertainment purposes, to mind the child during household errands, babysitting, etc.), whether the parents/guardians participated in these behaviors with their toddler, etc.

#### Participant characteristics

Parents/guardians of participating children completed a demographic questionnaire, which was distributed in the study package along with the letter of information and consent form. This questionnaire solicited data on toddlers’ sex, age, ethnicity, childcare enrollment status, as well as various family variables (e.g., annual family income, family status, parental education, etc.).

### Statistical analysis

Accelerometer data were downloaded using Actical-specific software (version 3.10). Comparable to the procedures described by Esliger, Copeland, Barnes, and Tremblay [33] and Esliger and Tremblay [34], the raw activity data were analyzed using custom software *KineSoft* version 3.3.62 (KineSoft, Loughborough, UK) to generate a series of standardized outcome variables. Consistent with Van Cauwenberghe and colleagues’ [14] process, decision rules from the preschool literature were used to reduce the collected toddlers’ accelerometry data. Specifically, non-wear-time was defined as 60 min of consecutive zeroes (which was cross-referenced with participants’ wear-time logs) and only participants who accumulated at least 4 valid days (3 weekdays and 1 weekend day; with a minimum wear time of 8 h per day) were retained for analysis. Naps were considered non-wear time. Participants not meeting this requirement were removed from the data set (*n* = 7). As a result, 85.1 % (i.e., 40/47) participants passed these quality control criteria, and were thus retained for all analyses.

*KineSoft* was used to compare the accelerometry data against Trost and colleagues’ [35] toddler- and device-specific cut-points (sedentary time [≤114 counts⋅15 s^−1^⋅epoch^−1^], LPA [≥115 ≤697 counts⋅15 s^−1^⋅epoch^−1^], and MVPA [≥698 counts⋅15 s^−1^⋅epoch^−1^], and TPA [≥115 counts⋅15 s^−1^⋅epoch^−1^]) to determine the amount of activity accumulated at various intensity levels – this was achieved by entering the cut-points into the program and then processing the included data files to produce a number of outcome variables using these thresholds. Because the toddler population has only recently begun to receive attention regarding physical activity levels, combined with the evidence that suggests that different accelerometers and/or their respective cut points can influence the outcome data (i.e., physical activity levels), it was deemed important to apply a second set of cut-points (used by other researchers who included toddler participants) for comparison. As such, and in line with the Canadian Health Measures Survey (CHMS), the following cut-points (all divided by four to match the time sampling interval used in the present study) were also applied to the collected accelerometer data : sedentary activity (≤24.75 counts⋅15 s^−1^) [36], LPA (≥25 ≤287.25 counts⋅15 s^−1^⋅epoch^−1^), and MVPA (≥287.5 counts⋅15 s^−1^⋅epoch^−1^), and TPA (≥25 counts⋅15 s^−1^⋅epoch^−1^) [37].

The data provided in *KineSoft*’s output report were transferred to SPSS (version 22) for descriptive analyses (means and standard deviations). To account for variances in monitoring periods, activity variables were reported as hourly rates (mins/hr) and percent of wear-time. Similar to the approach undertaken by Colley *et al.* [6], participants were classified as meeting the physical activity guidelines if they achieved 180 min of activity at any intensity on all valid days. Independent samples *t*-tests were conducted to explore whether toddlers’ rates of physical activity and sedentary time differed based on sex and childcare enrolment (i.e., yes/no; where children who attended home- and center-based care were combined). Paired samples *t*-tests were also carried out to explore whether this group’s activity levels differed based on cut-points and between weekdays and weekend days. Consequently, for the paired samples *t*-test, an alpha was adjusted to account for per comparison bias (0.05/2). Linear regression analyses were also carried out to explore the relationship between sedentary time and physical activity (all intensities; using both sets of cut-points) and multiple variables like sex, childcare attendance, parental education, annual family income, and total screen-viewing on weekdays/weekend days.

Descriptive analyses were conducted to evaluate the findings from the Toddler Screen-Viewing Questionnaire. Linear regression was used to examine potential associations between toddlers’ levels of sedentary time and parent-reported screen-viewing behaviors (i.e., *does your child watch television? [how many minutes per week(end) day?], and does your child spend time on a computer? [how many minutes per week(end) day?*]). To determine the number of participants that met/failed to meet the screen-use portion of the sedentary behavior guidelines (i.e., no screens for children under the age of 2, and limited to 1 hour per day for children 2–4 years), an approach undertaken by other Canadian researchers was followed [6]. Specifically, the mid-points of the previous categories were used to derive time spent watching television and using the computer on both weekdays and weekend days (i.e., 0, 15, 45, 75, 105, and 120mins). The amount of time on weekdays and weekend days were summed for the related questions to ascertain whether participants were meeting/failing to meet screen-time recommendations. Please refer to Colley *et al.* [6] for additional details regarding this process.

## Results

### Sample description

Demographic characteristics of the 40 toddlers included in the study are presented in Table 1. The average age of the sample was 25.7 months (*SD* = 5.9) and 55.0 % of the sample was female. The included sample’s mean accelerometry wear-time for valid days was 606.79 min (*SD* = 38.76) or 10.11 h, and ranged from 536.50 to 731.70 min.

**Table 1:** Toddler and family demographic information (*N* = 40)

	Number	Percent
Sex of toddler		
Male	18	45.0
Female	22	55.0
Type of early learning environment		
Home-based childcare	7	17.5
Center-based childcare	17	42.5
Other	2	5.0
Not in care	14	35.0
Ethnicity		
Caucasian	35	87.5
Latin American	1	2.5
Asian	1	2.5
Other	2	5.0
Family situation		
Single-parent	2	5.0
Double-parent	38	95.0
Highest level of Parent/Guardian education		
College	8	20.0
University	13	32.5
Graduate school	17	42.5
Prefer not to answer	1	2.5
Approximate annual household income		
Less than $20,000	2	5.0
$20,000 – $39,999	4	10.5
$40,000 – $59,999	3	7.5
$60,000 – $79,999	4	10.0
$80,000 – $99,999	4	10.0
$100,000 – $119,999	7	17.5
$120,000 – $149,000	4	10.0
More than $150,000	9	22.5
Prefer not to answer	3	7.5

Demographic information is reported for participants who provided sufficient physical activity data (i.e., a minimum of 4 valid days, with 8 h of data/day) – 3 participants did not meet these criteria, and were therefore removed. All values shown may not add up to 100 % or *n* = 40 as some individuals chose not to answer certain questions

### Toddlers’ levels of sedentary time and physical activity

**Fig. 1:**
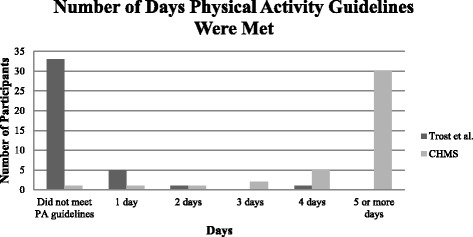
Number of days physical activity guidelines were met

Refer to Table 2 for toddlers’ sedentary and physical activity rates. Specifically, sedentary time ranged from 37.27 to 49.40 mins/hr, LPA from 9.79 to 18.78 mins/hr, MVPA from 0.82 to 3.95 mins/hr, and TPA from 10.60 to 22.73 mins/hr. Rates of sedentary time (*t*[39] = 37.81, *p* <.001), LPA (*t*[39] = −21.99, *p* <.001), MVPA (*t*[39] = −14.87, *p* <.001), and TPA (*t*[39] = −37.81, *p* <.001) were found to significantly differ based on cut-points applied. Using an average wear-time of 10.11 h, these values translate roughly to 376.80 and 499.43 mins/day of sedentary time, 98.97 and 189.87 mins/day of LPA, 8.29–39.93 mins/hr of MVPA, and 107.17–229.80 mins/hr of TPA when Trost *et al.* [35] and the CHMS [36, 37] cut-points were applied, respectively. Seven participants (i.e., 17.5 % of sample) met and/or exceeded the Canadian physical activity guidelines on at least one valid day when Trost *et al.*’s [35] cut-points were applied, whereas 39 participants (i.e., 97.5 % of sample) met/exceeded these guidelines when the CHMS [36, 37] cut-points were used. Figure 1 displays the number of days that participants met/exceeded the daily physical activity recommendations.

**Table 2:** Toddlers’ mean (SD) physical activity and sedentary time (Mins/Hr and Percentage of Monitoring Time) based on different cut-points

	Intensity		Trost *et al.*	CHMS^a^
			Mean (*SD*)	Mean (*SD*)
Combined (*n* = 40)	Sedentary	Mins/Hr	49.40 (3.29)*	37.27 (3.97)*
		% wear time	82.33 (5.49)	62.12 (6.62)
	LPA	Mins/Hr	9.79 (2.90)*	18.78 (3.22)*
		% wear time	16.31 (4.83)	31.30 (5.37)
	MVPA	Mins/Hr	0.82 (0.72)*	3.95 (1.93)*
		% wear time	1.36 (1.20)	6.59 (3.22)
	TPA	Mins/Hr	10.60 (3.29)*	22.73 (3.97)*
		% wear time	17.67 (5.49)	37.88 (6.62)
Male (*n* = 18)	Sedentary	Mins/Hr	48.93 (3.85)	37.25 (3.85)
		% wear time	81.56 (6.41)	62.09 (6.41)
	LPA	Mins/Hr	10.09 (3.31)	18.39 (3.00)
		% wear time	16.82 (5.52)	30.64 (5.01)
	MVPA	Mins/Hr	0.98 (0.90)	4.36 (2.38)
		% wear time	1.62 (1.50)	7.27 (3.97)
	TPA	Mins/Hr	11.07 (3.85)	22.74 (3.85)
		% wear time	18.44 (6.41)	37.91 (6.42)
Female (*n* = 22)	Sedentary	Mins/Hr	49.78 (2.80)	37.28 (4.16)
		% wear time	82.96 (4.66)	62.14 (6.94)
	LPA	Mins/Hr	9.54 (2.57)	19.10 (3.42)
		% wear time	15.89 (4.28)	31.83 (5.70)
	MVPA	Mins/Hr	0.69 (0.52)	3.62 (1.44)
		% wear time	1.15 (0.87)	6.03 (2.40)
	TPA	Mins/Hr	10.22 (2.80)	22.72 (4.16)
		% wear time	17.04 (4.66)	37.86 (6.94)

*CHMS* Canadian health measures survey, *LPA* Light-intensity physical activity, *MVPA* Moderate- to vigorous-intensity physical activity, *TPA* Total physical activity, *SD* Standard deviation, ^a^ Wong *et al.* [34] for sedentary cut-point and Adolph *et al.* [35] for MVPA cut-points. Thresholds for LPA were derived by researchers using the sedentary and MVPA cut-points. No significant differences in levels of physical activity and sedentary time based on sex were reported (*p* >.05). * A statistically significant difference was apparent between activity levels using the two different cut-points (*p* <.001)

While males accumulated less sedentary time and more MVPA and TPA (but not LPA) than their female counterparts, independent samples *t*-tests did not report any statistically significant differences in sedentary time (*t*[38] = −.082, *p* = .43), LPA (*t*[38] = 0.60, *p* = .55), MVPA (*t*[38] = 1.21, *p* = .24), or TPA (*t*[38] = 0.80, *p* = .43) based on the Trost *et al.* cut-points [35]. Likewise, when using the thresholds employed in the CHMS [36, 37]; sedentary time (*t*[38] = −.02, *p* = .98), LPA (*t*[38] = 0.69, *p* = .49), MVPA (*t*[38] = 1.16, *p* = .26), and TPA (*t*[38] = 0.02, *p* = .98) did not significantly differ based on sex.

Childcare attendance was only found to have a statistically significant effect on participants’ rates of LPA (CHMS cut-points only: *t*[36] = 3.07, *p* = .004). When comparing weekdays to weekend days, it was found that toddlers’ rates of sedentary time (*t*[39] = 17.11, *p* <.001), LPA (*t*[39] = 13.61, *p* <.001), MVPA (*t*[39] = 5.14, *p* <.001), and TPA (*t*[39] = 12.78, *p* <.001) were statistically significantly higher during the week than on the weekends using Trost *et al.* cut-points [35]. Similar statistically significant trends were noted for rates of sedentary time (*t*[39] = 14.80, *p* <.001), LPA (*t*[39] = 17.34, *p* <.001), MVPA (*t*[39] = 8.48, *p* <.001), and TPA (*t*[39] = 16.15, *p* <.001) using CHMS [36, 37] cut-points.

Linear regression analyses exploring the impact of sex, childcare attendance, screen viewing, and parental factors (income and education) on sedentary time and physical activity are presented in Tables 3 (Trost *et al.* cut-points) and 4 (CHMS cut-points) Overall, only those models using activity rates derived using the CHMS cut-points were statistically significant (*p* <.05).

**Table 3:** Summary of coefficients, *t*-values, *p*-values, and partial correlations for toddlers’ sedentary time and physical activity using Trost *et al.* cut-points

	Variable	B	*t*	*p*	Partial correlations
Sedentary time	Sex	0.64	0.63	0.54	0.11
	Childcare attendance	0.92	1.00	0.33	0.17
	Annual family income	0.30	1.40	0.17	0.24
	Parental education	0.51	0.79	0.44	0.14
	Total SV on weekdays	0.05	1.80	0.08	0.30
	Total SV on weekends	−0.07	−2.58	0.02	−0.41
LPA	Sex	−0.37	−0.40	0.69	−0.07
	Childcare attendance	−0.89	−1.08	0.29	−0.19
	Annual family income	−0.25	−1.31	0.20	−0.23
	Parental education	−0.45	−0.78	0.44	−0.14
	Total SV on weekdays	−0.06	−2.10	0.04	−0.35
	Total SV on weekends	0.06	2.51	0.02	0.41
MVPA	Sex	−0.27	−1.41	0.17	−0.24
	Childcare attendance	−0.03	−0.16	0.87	−0.03
	Annual family income	−0.05	−1.21	0.23	−0.21
	Parental education	−0.06	−0.48	0.64	−0.08
	Total SV on weekdays	0.00	0.37	0.71	0.07
	Total SV on weekends	0.01	1.65	0.11	0.28
TPA	Sex	−0.64	−0.63	0.54	−0.11
	Childcare attendance	−0.92	−1.00	0.33	−0.17
	Annual family income	−0.30	−1.40	0.17	−0.24
	Parental education	−0.51	−0.79	0.44	−0.14
	Total SV on weekdays	−0.05	−1.80	0.08	−0.30
	Total SV on weekends	0.70	2.56	0.02	0.41

Model accounts for 11.9, 9.5, 29.3 and 11.9 % of the variability in toddlers’ sedentary time, LPA, MVPA, and TPA, respectively

*LPA* Light-intensity physical activity, *MVPA* Moderate- to vigorous-intensity physical activity, *TPA* Total physical activity, *SV* Screen-viewing

**Table 4:** Summary of coefficients, *t*-values, *p*-values, and partial correlations for toddlers’ sedentary time and physical activity using the CHMS cut-points

	Variable	B	*t*	*p*	Partial correlations
Sedentary time	Sex	−0.24	−0.21	0.84	−0.04
	Childcare attendance	1.54	1.46	0.16	0.25
	Annual family income	0.20	0.82	0.42	0.14
	Parental education	0.67	0.90	0.38	0.16
	Total SV on weekdays	0.10	2.96	0.01	0.46
	Total SV on weekends	−0.10	−3.26	0.00	−0.50
LPA	Sex	0.87	0.94	0.35	0.16
	Childcare attendance	−1.24	−1.49	0.15	−0.26
	Annual family income	−0.01	−0.04	0.97	−0.01
	Parental education	−0.43	−0.73	0.47	−0.13
	Total SV on weekdays	−0.09	−3.16	0.00	−0.49
	Total SV on weekends	0.06	2.59	0.01	0.42
MVPA	Sex	−0.62	−1.15	0.26	−0.20
	Childcare attendance	−0.30	−0.62	0.54	−0.11
	Annual family income	−0.20	−1.70	0.10	−0.29
	Parental education	−0.24	−0.70	0.49	−0.12
	Total SV on weekdays	−0.02	−1.04	0.31	−0.18
	Total SV on weekends	0.04	2.64	0.01	0.42
TPA	Sex	0.24	0.21	0.84	0.04
	Childcare attendance	−1.54	−1.46	0.16	−0.25
	Annual family income	−0.20	−0.82	0.42	−0.14
	Parental education	−0.67	−0.90	0.38	−0.16
	Total SV on weekdays	−0.10	−2.96	0.01	−0.46
	Total SV on weekends	0.10	3.26	0.00	0.50

Model accounts for 19.4, 22.7, 25.7 and 19.4 % of the variability in toddlers’ sedentary time, LPA, MVPA, and TPA, respectively

*LPA* Light-intensity physical activity, *MVPA* Moderate- to vigorous-intensity physical activity, *TPA* Total physical activity, *SV* Screen-viewing

### Screen-viewing among toddlers

**Fig. 2:**
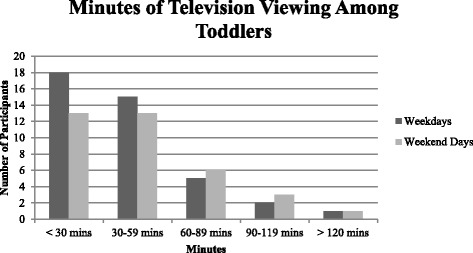
Minutes of television viewing among toddlers

**Fig. 3:**
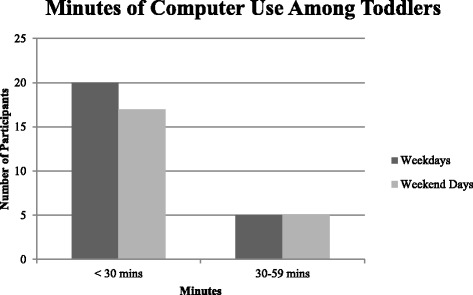
Minutes of computer use among toddlers

Descriptive statistics from the screen-viewing questionnaire revealed that 93.2 % of participants watched television (Fig. 2), while 56.8 % of participants utilized computers (which included laptops, tablets, and smartphones; Fig. 3). Only 6.82 % of parents/guardians reported that their toddler did not engage in any form of screen-based activity (i.e., did not watch television and did not use computers on weekdays or weekend days).

When asked what the main reasons (i.e., check all that apply) for why their toddler engaged in screen-viewing activities, parents/guardians indicated: 52.3 % for educational purposes, 65.9 % for entertainment purposes, 70.5 % to occupy the child while completing household errands, and 6.8 % during babysitting/childcare minding hours. Of those who responded, approximately 18.2 % of parents/guardians indicated that they *always* sit with their child while he/she watches television, while 68.2 and 4.5 % responded that they *sometimes* or *never* sit with their child while he/she watches television, respectively. Only 9 % of parents/guardians reported that the television is *always* left on in the background while their child plays; 47.7 and 43.2 % reported that it was *sometimes* or *never* left on in the background, respectively.

Regression analyses revealed that *television viewing* significantly predicted toddlers’ sedentary time using the CHMS cut-points (*F*[2, 33] = 5.27, *p* = 0.01, adj R^2^ = .01), but not those by Trost *et al.* (*F*[2, 33] = 2.13, *p* = 0.14, adj R^2^ = .06). Upon examination of the unique contributions to this model (and based on the CHMS thresholds), it was found that *television viewing* significantly predicted 48.7 % (*r* = 0.487, *p* <.001) and −47.9 % (*r* = −0.479, *p* <.001) of the variation in sedentary time on weekdays and weekend days, respectively. *Computer use* was not found to significantly predict sedentary time based on either set of cut-points (Trost *et al.*: *F*[1, 19] = 0.22, *p* = .64, adj *R*^2^ = −.04; CHMS: *F*[1, 19] = .27, *p* = .61, adj *R*^2^ = −.04).

When considering the Canadian sedentary behavior guidelines, only 18.8 and 25.0 % of children under 2 years and 70.8 and 62.5 % of 2–3 years olds met the screen-use recommendation of the sedentary behavior guidelines, on weekdays and weekend days, respectively.

## Discussion

This is the first Canadian study tasked with objectively measuring full-day physical activity and sedentary time among toddlers, with consideration to different cut-points, various demographic variables (i.e., sex, childcare enrollment, parental income and education), and weekday/weekend day variation. While levels of LPA, MVPA, and TPA were significantly variable (contingent on cut-points used; i.e., 9.79–18.78 mins/hr, 0.82–3.95 mins/hr, and 10.60–22.73 mins/hr mins/hr, respectively), sedentary levels were high among this sample (i.e., 37.27–49.40 mins/hr). Overall, it was found that in comparison to the CHMS cut-points [36, 37], the toddler-specific thresholds derived by Trost *et al.* [35] yield lower levels of LPA, MVPA, and TPA as well as higher levels of sedentary time.

By applying Trost *et al.*’s [35] cut-points, the findings reveal that the majority (i.e., 82.5 %) of toddlers are insufficiently active to meet current national physical activity guidelines. Interestingly, when the cut-points used in the CHMS were applied [36, 37] to the activity data, it was found that 97.5 % of participants met the physical activity guidelines on 1 or more days. Consequently, these findings highlight the challenges of accurately interpreting Canadian toddlers’ activity levels. Despite this large difference in adherence to national standards, this discrepancy may not be surprising given how much lower the CMHS cut-points [36, 37] are in comparison to those by Trost *et al.* [35]; consequently, many more minutes of collected data were likely classified as LPA rather than sedentary time. Nonetheless, regardless of the inconsistency in time spent in LPA, what may prove challenging in the future from a public health perspective, is that, regardless of which cut-points were applied, toddlers in the present study accumulated very little MVPA. While current guidelines for young children do not stipulate that physical activity at a particular intensity must be achieved [15], higher intensity activities will become increasingly important once children reach 5 years of age [38].

In line with the findings using Trost *et al.*’s [35] cut-points, low levels of physical activity have been echoed elsewhere in the literature among toddlers in other developed countries [7]. Specifically, Manios [7] reported that participants spent very little time in light to vigorous physical activity (via proxy questionnaire; 12–24 months: 1.45 ± 3.15 h/week for males and 1.05 ± 2.29 h/week for females; 25–36 months: 1.51 ± 2.63 h/week for males and 1.21 ± 2.41 h/week for females). During childcare hours, and consistent with the noted trends of this work, researchers have also reported that sedentary levels are high among this population [9, 13, 14]. The findings by Carson *et al.* [13] mirror very closely the LPA (i.e., 18.1 mins/hr) and sedentary levels (i.e., 37.8 mins/hr) of the toddlers in the current study.

The low levels of MVPA among participating toddlers were similar to Gubbels *et al.*’s [8] (where 5.5 % of indoor observations and 21.2 % of outdoor observations were classified as MVPA as directly observed via the Observational System for Recording Physical Activity in Children–Preschool Version; mean age = 2.6 years) and Witjzes *et al.*’s [11] (where 4.8 and 5.2 % of objectively monitored time via ActiGraph accelerometers was reported as MVPA on weekdays and weekend days, respectively) work also reported time spent in MVPA (albeit low) among their toddler samples. Young children from Carson *et al.*’s paper also reported some levels of MVPA (i.e., 4.0 mins/hr) during childcare hours using Actical accelerometers [13]. Participants in Hnatiuk and colleagues’ [10] (mean age = 19.1 [*SD* = 2.3] months) and Johansson and colleagues’ [12] (mean age = 2.03 [*SD* = 0.1] years) research participated in slightly higher levels of MVPA; 47 and 84 mins/day (measured via ActiGraph accelerometers), respectively.

Discrepancies in values observed across studies could be a result of measurement differences encountered using ActiGraph versus Actical accelerometers, and their associated cut-points [26]. If fact, a recent paper by Vanderloo *et al.* [26] found that in comparison to Actical accelerometers, ActiGraph accelerometers report higher levels of physical activity and lower levels of sedentary time among young children. Further to this point, and specific to the toddler population, the cut-points derived by Trost *et al.* differ significantly for Actical [35] (used in the present study) and ActiGraph [39] (used in previous studies [10–12]) devices using 15 s epochs: 0–114 counts versus 0–48 counts for sedentary time, 115–697 counts versus 49–418 counts for LPA, and >697 counts versus >418 counts for MVPA; respectively. Another possible explanation for the lower levels of MVPA accumulated by this sample may be the choice of cut-points applied to this data. To the authors’ knowledge, the cut-points derived by Trost and colleagues [35] are the only thresholds that have been identified for use with Actical accelerometers and toddlers. It is possible that the cut-points used to interpret the activity data may have resulted in the misclassification of MVPA into LPA and/or of LPA into sedentary time. As such, additional validation work is needed to develop universally accepted cut-points that define various intensity levels among toddlers. To further investigate this issue, researchers employed a similar method to Colley and colleagues’ [6] cross-sectional investigation of preschoolers’ physical activity levels (who reported MVPA levels ranging from 17 to 68 min depending on cut-points used), and applied a second set of cut-points [36, 37] to the data in order to explore differences in activity levels. Evidently, these findings may draw attention to the fact that accelerometers *alone* may not provide a complete picture of toddlers’ physical activity behaviors; additional contextual information is needed to help subsidize the objective data.

Comparable to Gubbel *et al.*’s [8], Fees *et al.*’s [9], Hnatiuk *et al.*’s [10], and Johannson *et al.*’s [12] work, but in contrast to Witjzes *et al.*’s [11], levels of physical activity did not significantly differ based on sex. Interestingly, while the impact of sex on toddlers’ physical activity levels may not be entirely clear, it is possible that this biological factor may play a greater role in children’s activity behaviors as they age (i.e., preschool- and school-age years). While not overly unexpected that the toddlers in this study accumulated low levels of physical activity (depending on the cut-points applied), it was somewhat surprising to see such low numbers among a sample where the majority were from families with higher socio-economic statuses (SES; where higher SES has been linked to higher rates of physical activity among children [40]). This finding may suggest that even toddlers from higher income homes are not immune to inactivity.

Participants from this study were found to engage in high levels of sedentary time (i.e., approximately 81.72 and 62.54 % of monitoring time based on Trost *et al.* and CHMS cut-points, respectively). Given the many negative health outcomes associated with sedentary behaviors [41], these findings are alarming and unfortunately, not unique. Gubbels and colleagues [8] (where approximately 59.4 % of the indoor and 31.2 % of the outdoor observations were classified as sedentary), Johansson *et al.* [12] (where approximately 55 % of monitoring time was sedentary), and Witjzes and colleagues [11] (where approximately 85 % of monitoring time on both weekdays and weekend days were sedentary) also reported high levels of sedentary time among their toddler samples. Witjzes *et al.* [11] also reported that female toddlers engaged in significantly more sedentary time than their male counterparts; however, this was not the case in the present study.

One behavior that might account for a large proportion of this sample’s sedentary time could be their high levels of television viewing and computer use. This paper marks one of the first explorations of screen-viewing among toddlers in Canada and revealed that on weekdays and weekend days respectively, 81.2 and 75.0 % of children under 2 years and 29.2 and 37.5 % of 2–3 years olds failed to adhere to the screen-use portion of Canada’s sedentary behavior guidelines for young children. Similarly, a brief review by Cardon *et al.* [24] found that screen use is very common among young children; these findings are concerning as it is possible that screen-viewing time may be displacing physical activity (particularly at light intensities) [42]. Unfortunately, our finding that toddlers are spending large amounts of time viewing screens aligns with the research-based recognition that next to sleeping, the time children spend engaged in screen-viewing exceeds that of any other in which they would typically participate [43]. Consequently, given current guidelines which recommend that young children should not spend more than 60 min in a single bout of sitting and or restrained [16], combined with the fact that as sedentary behaviors tend to persist throughout the lifespan [44], increased research efforts are also needed to address *why* toddlers are spending significant amounts of time engaging in screen-viewing activities during this critical developmental period. Garnering such information would prove useful in developing and instilling mechanisms to help parents limit their toddlers’ engagement in screen-viewing activities.

Due to the young age of the participants, compliance in wearing the belts throughout the entire data collection period was, at times, challenging (as noted by parents/guardians in the wear-time logs). Despite this, the majority of participants had adequate wear time to be included in all analyses. Future research with toddlers may also consider defining non-wear time as 20 min of consecutive zeros (rather than 60 min) as it may be more reasonable to consider this age group remaining still for 20 min (rather than 60 min). Although efforts were made to achieve a geographically-representative sample, the generalizability of these results may be limited by the small sample size used. This is the first study to apply the Trost and colleagues [35] cut-points to Actical accelerometer data, which makes comparisons with previous studies challenging. However, given that these are the only available cut-points that are both toddler- and Actical-specific, the authors felt it was important to utilize these thresholds in the present paper. Lastly, while the Toddler Screen-Viewing Questionnaire was informed by previous studies [6, 22, 23, 32], its psychometric properties have not been assessed, and as such, its validity has not been established.

## Conclusion

The findings from this work highlight the challenge of accurately interpreting toddlers’ levels of physical activity an sedentary time, which consequently makes comparisons to national guidelines challenging. In comparison to the CHMS cut-points [36, 37], it was found that the toddler-specific cut-points derived by Trost *et al.* [35] produce much lower levels of physical activity and higher levels of sedentary time. Despite this noted challenge, this study highlights the high levels of sedentary behaviors in which toddlers are participating – this aligns with previous studies with this population. Finally, our work presents the first depiction of screen-viewing behaviors, and their alignment with national standards among this young cohort. In light of the growing interest in toddlers’ physical and sedentary behaviors, additional research is required to confirm these findings as well as to explore mechanisms for promoting active behaviors among this group (and minimizing sedentary ones) to ensure healthy growth and development.

## Abbreviations

CHMS: Canadian health measures survey
LPA: Light-intensity physical activity
MVPA: Moderate- to vigorous-intensity physical activity
TPA: Total physical activity
